# Role of myosin light chain kinase in intestinal epithelial barrier defects in a rat model of bowel obstruction

**DOI:** 10.1186/1471-230X-10-39

**Published:** 2010-04-20

**Authors:** Chi-Chin Wu, Yen-Zhen Lu, Li-Ling Wu, Linda C Yu

**Affiliations:** 1 Graduate Institute of Physiology, National Taiwan University College of Medicine, Taipei, Taiwan

## Abstract

**Background:**

Bowel obstruction is a common cause of abdominal emergency, since the patients are at increased risk of septicemia resulting in high mortality rate. While the compartmentalized changes in enteric microfloral population and augmentation of bacterial translocation (BT) have already been reported using experimental obstruction models, alterations in epithelial permeability of the obstructed guts has not been studied in detail. Myosin light chain kinase (MLCK) is actively involved in the contraction of epithelial perijunctional actinomyosin ring and thereby increases paracellular permeability. In the current study we attempt to investigate the role of MLCK in epithelial barrier defects using a rat model of simple mechanical obstruction.

**Methods:**

Wistar rats received intraperitoneal injection of ML-7 (a MLCK inhibitor) or vehicle at 24, 12 and 1 hrs before and 12 hrs after intestinal obstruction (IO). The distal small intestine was obstructed with a single ligature placed 10 cm proximal to the ileocecal junction in IO rats for 24 hrs. Sham-operated rats served as controls.

**Results:**

Mucosal injury, such as villous blunting and increased crypt/villus ratio, was observed in the distal small intestine of IO rats. Despite massive enterocyte shedding, intestinal villi were covered with a contiguous epithelial layer without cell apoptosis. Increased transmural macromolecular flux was noticed in the distal small intestine and the proximal colon after IO. The bacterial colony forming units in the spleen and liver of IO rats were significantly higher than those of sham controls. Addition of ML-7 ameliorated the IO-triggered epithelial MLC phosphorylation, mucosal injury and macromolecular flux, but not the level of BT.

**Conclusions:**

The results suggest that IO-induced premature enterocytic sloughing and enhanced paracellular antigenic flux were mediated by epithelial MLCK activation. In addition, enteric bacteria may undergo transcytotic routes other than paracellular paths to cross the epithelium.

## Background

Bowel obstruction is one of the most common causes of abdominal emergency. Single or multiple sites of obstructions are found in patients with post-operative adhesions, incarcerated hernia, gut strangulation, intussusception, and abdominal tumors [1-3]. Patients with mechanical bowel obstruction are of increased risk of septicemia which results in high rates of mortality [1,2]. The septic complications are associated with gut barrier dysfunction, and entry of gut microorganisms and bacterial products [4,5].

Intestinal lumen hosts a large amount of commensal bacteria that is normally separated from the body proper by epithelial cells linked by tight junctions [6,7]. The highest number of bacteria is found in the colon with a gradual decrease in upper bowel segments. In obstructed guts, massive quantity of bacteria and antigens penetrates into extraintestinal organs, which is termed bacterial translocation (BT) [4,5,8-10]. Changes in gut microfloral population have been implicated in the mechanism of BT. In a murine model of single-site bowel obstruction, the bacterial population in various gut segments multiplied 100 to 1000 folds [8]. Interestingly, bacterial overgrowth was more pronounced in the ileum compared to the cecum or colon [8]. Aside from the phenomena of BT and compartmentalized bacterial overgrowth, limited information exists on changes of epithelial permeability in obstructed guts.

The epithelial tight junctions (TJ) are regulated by the contraction of perijunctional actinomyosin ring. The phosphorylation of myosin light chain (MLC) by myosin light chain kinase (MLCK) results in increased paracellular permeability [11-13]. The phenomena of MLC phosphorylation and TJ reorganization are also involved in epithelial shedding at villous tips, contributing to normal cell turnover along the crypt-villus axis [14]. Physiological extrusion of enterocytes does not compromise the epithelial barrier function. However, a rise in intestinal permeability caused by MLCK activation was demonstrated in numerous disease models, including endotoxemia, pro-inflammatory stress, giardiasis and T cell-activated colitis [15-17]. Whether bowel obstruction could induce antigenic flux and BT via activation of epithelial MLCK has not been examined so far.

Hence we determined to investigate the compartmentalized changes of epithelial permeability, and to assess the role of MLCK in abnormal epithelial shedding, macromolecular influx and BT in obstructed guts, using a rat model of simple mechanical obstruction.

## Methods

### Animals

Male Wistar rats aged 6 to 9 weeks and weighing 200-300 g were used for the study. Animals were raised in a temperature-controlled room (20 ± 2°C) with 12-hr light-dark cycles, and fed with regular rat chow and water. The experimental procedures were approved by the Laboratory Animal Care Committee, National Taiwan University College of Medicine.

### Experimental design

In the first set of experiments, rats were randomly divided to sham-operation (sham), and intestinal obstruction (IO) groups (n = 5-7/group). The experimental protocols were carried out under aseptic conditions. After anesthetizing with sodium pentobarbital (50 mg/kg, intraperitoneal (i.p.) injection), rats underwent midline laparotomy. In IO rats, the distal small intestine was obstructed with a single ligature of 4-0 silk placed 10 cm proximal to the ileocecal junction. Sham-operated rats received mock manipulation of the gut without ligation. In all animals, care was taken not to occlude or puncture mesenteric vessels and no sign of cyanosis was seen during the experiment. The abdominal wall was sutured after surgery and the incision sterilized with povidone iodine. During these procedures, the arterial blood pressure, heart rate, and body temperature were closely monitored. The average operating time per animal was less than 15 minutes. The rats were placed into steel cages, fasted but with free access to water. Twenty-four hours later, sham and IO rats were sacrificed. In addition, ischemia/reperfusion (I/R) rats were used as positive controls to show epithelial discontinuity and villous denudation. The superior mesenteric artery was occluded with an artery clamp for 20 minutes and released for one hour for reperfusion. The gut tissues were collected at the end of the procedure.

In the next series of experiments, animals were randomized to four experimental groups (n = 5-7/group): group 1 (sham+vehicle); group 2 (IO+vehicle); group 3 (sham+ML-7), and group 4 (IO+ML-7). ML-7 (5-iodonaphthalene-1-sulfonyl, Sigma) is a selective MLCK inhibitor. ML-7 (1 mg/kg) or its vehicle (0.9% NaCl) was administered i.p. in a volume of 250 μl at 24, 12, and 1 hr before and 12 hrs after intestinal ligation or sham-operation. ML-7 was used at a dose previously described in rodent models to inhibit LPS- and stress-induced increase of intestinal paracellular permeability [15,16]. No bactericidal or bacteristatic activity was seen with ML-7 in our preliminary study. The animals received either sham-operation or IO were sacrificed 24 hrs after surgery. Two 2-cm intestinal segments from the duodenum (starting at 1 cm distal to the pylorus), jejunum (15 cm distal to the pylorus), distal small intestine (10 cm proximal to the ileocecal junction), proximal colon, and distal colon [18] were excised for experiments.

### Histopathological examination

Tissues were fixed in 4% paraformaldehyde and embedded. Sections of 4 μm thickness were stained and examined by light microscopy. The villous height and crypt depth were measured from well-oriented, complete crypt-villous units using a scale built in the optical lens of the microscope. Three-to-five crypt-villus units were measured in each rat sample. The average of the calculated ratio of crypt to villus was determined for each rat in the different groups [19].

Light microscopic studies were reviewed in a blinded manner using a histopathological scale previously described [20]. Briefly, the tissue damage was graded from 0 to 5 according to the following criteria: grade 0, normal structure of villi; grade 1, development of small subepithelial space at the villous apex; grade 2, enlarged subepithelial space but without change in villous length and width; grade 3, few shortened villi and presence of cells in the lumen; grade 4, the majority of villi are shortened and widened with crypt hyperplasia and cells in the lumen; grade 5, blunting of all villi with elongated crypts and a high number of cells in the lumen.

### TUNEL assay

DNA-strand breaks (the hallmark of cell apoptosis) were detected *in situ *by a terminal deoxynucleotide transferase biotin-dUTP nick-end labeling (TUNEL) method. A TdT-FragEL™ DNA fragmentation detection kit (Calbiochem) was utilized following the manufacturer's protocol. The number of TUNEL(+) cells was quantified in photomicrographs of intestines taken from three-to-five rats per group at 100× magnification.

### Ussing chamber studies and Permeability assay

Intestinal segments were removed and gently rinsed with PBS. Care was taken to avoid Peyer's patches. Muscle-stripped tissues were opened along the mesenteric border and mounted in Ussing chambers (WPI Instruments). The opening area (2 cm^2^) of the chamber exposed the tissue to 5 ml of circulating oxygenated Krebs buffer. The serosal buffer contained 10 mmol/L of glucose that was osmotically balanced with 10 mmol/L of mannitol in the mucosal buffer. The temperature of the buffer was maintained at 37°C using a circulating water bath. The tissues were clamped at 0 V using a voltage clamp. The potential difference (PD, mV) and the short-circuit current (Isc, μA/cm^2^) of the tissues were determined on line. The tissues were pulsed with 1 mV for a duration of one second in 5-minute intervals, and the change in the Isc caused by the pulse was used to calculate the tissue conductance (mS/cm^2^) according to Ohm's law [21,22].

The intestinal epithelial permeability was determined by the level of mucosal-to-serosal flux of horseradish peroxidase (HRP, type II, MW = 44 kD, Sigma). Tissues on the Ussing Chambers were allowed to equilibrate until the Isc stabilized before HRP was added to the luminal buffer at a final concentration of 5 × 10^-5 ^mol/L. Samples (300 μl) of serosal buffer were collected at 0, 30, 60 and 90 minutes after luminal addition of HRP, and were replaced with Krebs buffer. The concentration of HRP was determined by a kinetic enzymatic assay. Fluxes were calculated according to standard formulae and were expressed as pmol/cm^2^/hr [21,22].

### Analysis of bacterial translocation (BT)

Under sterile conditions, parts of the liver and spleen were removed and weighed. Each organ was homogenized and sonicated in 10 times volume of sterile PBS, and cultured on fresh blood agar plates (Scientific Biotech Corp.) at 37°C for 24 hours. The bacterial colonies were counted and normalized to colony-forming units per gram of tissue (CFU/g) [23].

### Measurement of luminal bacterial counts

The distal small intestine of 10-cm length, with thread ligation at one end, was removed after IO for 24 hrs. The intestinal loop was instilled with sterile PBS (0.25 ml) and rocked back and forth for 10 times. The intestinal lavage was plated on fresh blood agar overnight, and the number of bacteria was expressed as log colony-forming units per ml (log CFU/ml).

### Western Immunoblotting of phosphorylated MLC (pMLC)

Scraped mucosa was homogenized in 10 times volume of complete RIPA buffer and sonicated for 10 seconds. The lysate was centrifuged and the protein concentration of the supernatant was adjusted to 5 mg/ml. The supernatant was dissolved in 2× electrophoresis sample buffer. Samples were boiled in 95°C heat block, and stored at -20°C until the use for immunoblotting.

The samples were separated by 4-12% SDS/PAGE, and the resolved proteins were electrotransferred onto membranes. After blocking in 5% nonfat dry milk, the membrane was incubated with a polyclonal rabbit anti-human phospho-MLC antibody (1:200, Santa Cruz) or a monoclonal mouse anti-β-actin (1:2000, Sigma) at 4°C overnight [16]. Membranes were washed and incubated with goat anti-rabbit IgG (1:1000, Cell Signaling) and goat anti-mouse IgG (1:2000, Santa Cruz) for 1 hour. After additional washes, the antigens were revealed using chemiluminescence (ECL) detection reagents (Millipore). Band density was quantified by photoimage analysis.

### Immunofluorescent staining

Intestinal tissue sections were blocked and incubated with rabbit anti-phospho-myosin light chain 2 (Cell Signaling) overnight at 4°C. The sections were washed with PBS and incubated with Alexa Fluor^® ^488 goat anti-rabbit IgG (Invitrogen) for 1 hr. The sections were washed, and the cell nuclei were stained with a Hoechst dye (Invitrogen). Images were captured using a fluorescence microscope.

### ELISA

Scraped mucosa was homogenized and sonicated in PBS, and the lysate was centrifuged. The protein concentration in the supernatant was quantified. The levels of TNFα in intestinal mucosa and plasma samples were measured by using ELISA development kits (PeproTech) according to the manufacturer's instruction. The cytokine levels in intestinal mucosa were expressed in pg/mg of protein.

### Statistical analysis

All data except for bacterial CFU/g were presented as mean ± SEM. The data were compared by ANOVA, followed with Student-Newman-Keuls post-hoc test wherever appropriate (Sigma Stat). The median of CFU/g values were compared using the Mann Whitney nonparametric test. The villous length, crypt depth, crypt/villi ratio and histological damage score were compared using t-test. A value of p < 0.05 was considered statistically significant.

## Results

### Intestinal obstruction (IO) caused histological injury and inflammation in the distal small intestine

**Figure 1:**
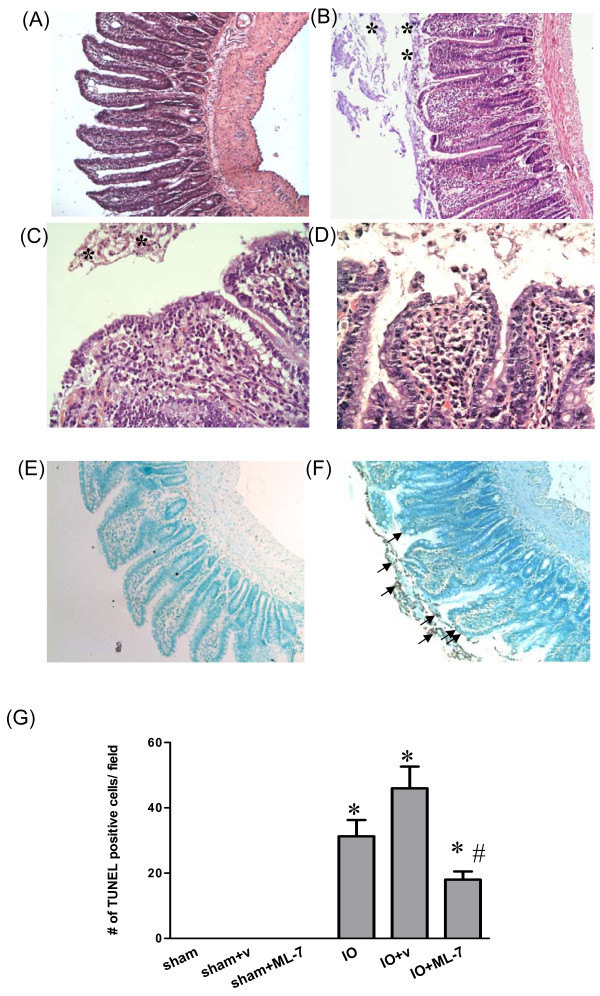
**Histological images of the distal small intestine of sham and IO rats**. **(Panels A-D) **Sections of intestinal tissues were stained with H & E. **(Panels E and F) **The apoptotic level of intestinal tissues was examined by TUNEL assay. **(A) **Normal villous structure was seen in the distal small intestine of sham rats. **(B) **Following IO, villi were edematous and blunted. Severe shedding of epithelial cells was observed in the intestinal lumen of IO rats (asterisk). **(C) **Higher magnification of the epithelial layer in the distal small intestine of IO rats showing epithelial shedding without villous denudation. **(D) **A positive control displaying epithelial discontinuity in an ischemic gut model. **(E) **No sign of TUNEL(+) cells was observed in the intestine of sham controls. **(F) **A high number of apoptotic cells was seen in the gut lumen following IO (arrows). **(G) **Quantification of TUNEL(+) cells. *p < 0.05 vs. respective sham groups. ^#^p < 0.05 vs. IO+v rats. Panels A, B, E and F: 100× magnification. Panels C and D: 400× magnification.

**Figure 2:**
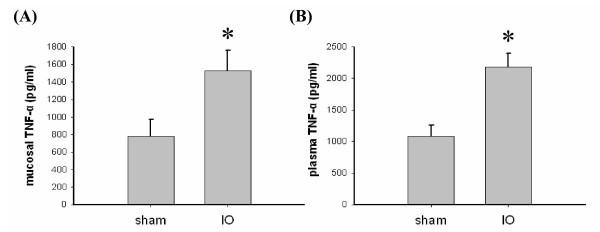
**Enhanced mucosal and plasma TNFα levels following IO**. (A) The TNFα levels in the mucosa of the distal small intestines in sham and IO rats. (B) The plasma TNFα concentration in sham and IO rats. n = 5/group. *p < 0.05 vs. sham rats.

In contrast to the normal villous structure in sham rats (Figure 1A), mucosal injury, such as villous blunting and epithelial sloughing, were observed in the distal small intestine of IO rats (Figure 1B). The crypt/villi ratio in distal small intestine of sham rats was significantly lower than that of IO rats (0.651 ± 0.023 vs. 1.124 ± 0.107, p < 0.05). The majority of the shortened villi were seen fully covered with a contiguous epithelial layer without surface denudation in IO rats (Figure 1B and 1C). A positive control showing villous epithelial discontinuity was previously established in ischemic gut models (Figure 1D). Increased cellularity and inflammatory infiltrate were seen in the lamina propria of IO rats. Moreover, the levels of TNFα in the intestinal mucosa and plasma samples of IO rats were higher than those of sham controls (Figure 2).

A higher number of TUNEL (+) apoptotic cells in the intestinal lumen was noticed in IO rats compared to sham controls (Figure 1E-G). Most apoptotic cells were present in the luminal area but not on the villous surface of IO rats (Figure 1F). The overall histopathological damage score in the intestine of sham and IO rats were statistically different (0.70 ± 0.30 vs. 4.50 ± 0.16, p < 0.05). The mucosal histology and cell apoptosis level in other segments of the intestine, including duodenum, jejunum, proximal and distal colon, were comparable between sham and IO rats (data not shown).

### Enhanced transepithelial permeability to ions and macromolecules was found in the distal small intestine and proximal colon after IO

**Figure 3:**
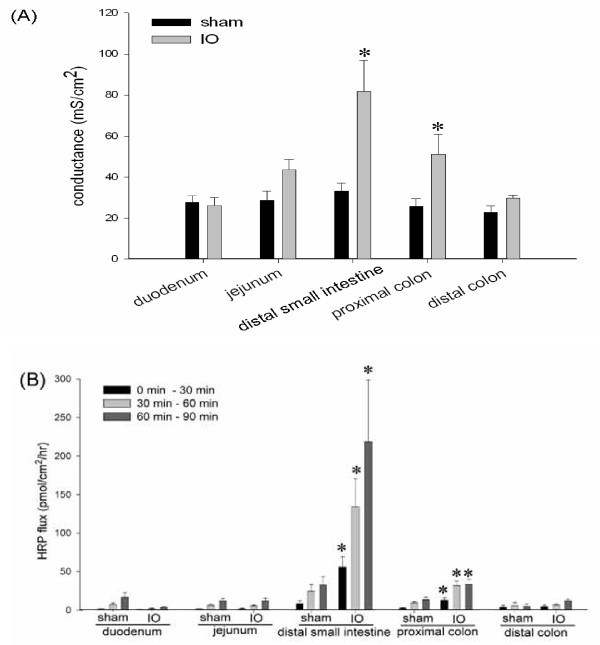
**IO induced epithelial permeability rise in the distal small intestine and proximal colon**. Various bowel segments in sham and IO rats were mounted on Ussing chambers for the measurement of electrical conductance **(Panel A) **and mucosal-to-serosal flux of HRP **(Panel B)**. The rate of HPR flux was determined at different time points after luminal addition of the probe: 0 to 30, 30 to 60, and 60 to 90 min. n = 5-7/group. *p < 0.05 vs. respective sham groups.

To investigate the effect of IO on epithelial barrier function, gut segments were collected for the measurement of transmural electrical conductance and HRP flux. The conductance of the distal small intestine in IO rats (81.69 ± 15.23 mS/cm^2^) was higher than that of sham controls (33.22 ± 3.74 mS/cm^2^) (Figure 3A). While increased tissue conductance was also found in the proximal colon of IO rats, the duodenum, jejunum and distal colon in IO rats were unaffected compared to sham controls (Figure 3A). The transmural flux rate of HRP was significantly higher in the distal small intestine of IO rats compared to their sham counterparts (Figure 3B). A statistically significant rise in HRP flux was also noticed in the proximal colonic tissues in IO rats (Figure 3B).

### IO triggered enteric bacterial overgrowth and bacterial translocation to the spleen and liver

**Figure 4:**
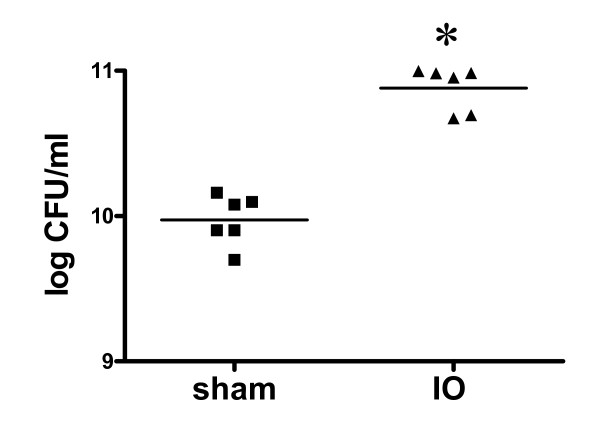
**Bacterial overgrowth in the small intestinal lumen in IO rats**. A tenfold increase of bacterial colony forming units (CFU) was seen in the intestinal lavage in IO rats compared to sham controls. Each data point in the figure represents the value from one animal, and the mean values of bacterial counts are shown as bars. n = 6/group. *p < 0.05 vs. sham rats.

A tenfold increase of the luminal bacterial counts was seen in the distal small intestine of IO rats compared to sham controls, suggesting enteric bacterial overgrowth following bowel obstruction (Figure 4). A significant increase in the mean bacterial CFU was found in the spleen of IO rats compared to that of sham rats (672.7 ± 222.3 vs. 10.0 ± 6.7 CFU/g, p < 0.05). The amount of viable bacteria in the liver was also higher in IO rats than in sham controls (300.0 ± 72.0 vs. 0.0 ± 0.0 CFU/g, p < 0.05). Due to the large variation among bacterial CFU numbers in each group, the median scores were analyzed by nonparametric statistical methods which showed similar results as well.

### Increased MLC phosphorylation was found in the intestinal epithelium of IO rats

**Figure 5:**
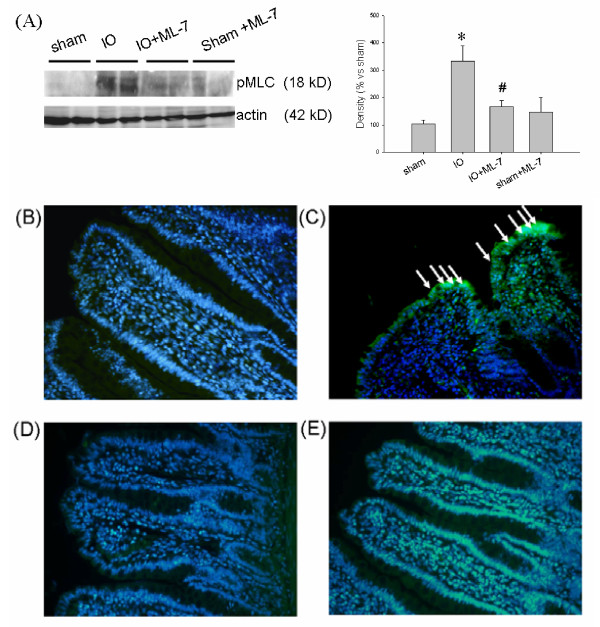
**Increased phosphorylation of myosin light chain in intestinal epithelium in IO rats was inhibited by treatment with ML-7**. **(A) **Western blots showing that IO augmented the mucosal levels of phosphorylated MLC (pMLC), which was attenuated by ML-7. n = 3-4/group. *p < 0.05 vs. respective sham rats. ^#^p < 0.05 vs. IO rats. Representative images of pMLC staining in intestinal tissues of **(B) **sham, **(C) **IO, **(D) **sham+ML-7, and **(E) **IO+ML-7 rats. The staining of pMLC (green color) was localized to the villous surface (arrows) in IO rats, whereas no staining was seen in sham controls. The cell nuclei were shown in blue color. Addition of ML-7 prevented the IO-induced phosphorylation of epithelial MLC. Images: 200× magnification.

A 3-fold increase in the level of phosphorylated MLC was seen in the intestinal mucosa of IO rats compared to sham controls (Figure 5A). Immunohistological staining confirmed the increase of pMLC on villous epithelium following IO (Figure 5B and 5C). No staining was observed in the villous core or muscular layer in the distal small intestine of IO rats ruling out the involvement of endothelial and smooth muscle cells which also contain MLC. Isotype antibody controls showed negative staining (data not shown). To decipher whether MLCK activation was involved in the intestinal permeability changes, rats were injected (i.p.) with ML-7 (an inhibitor to MLCK) or vehicle (v) before operation. Treatment with ML-7 reduced the increase of pMLC levels in the intestinal epithelium caused by IO (Figure 5A-E).

### Treatment with ML-7 alleviated mucosal injury, and prevented the rise of gut permeability in IO rats

**Figure 6:**
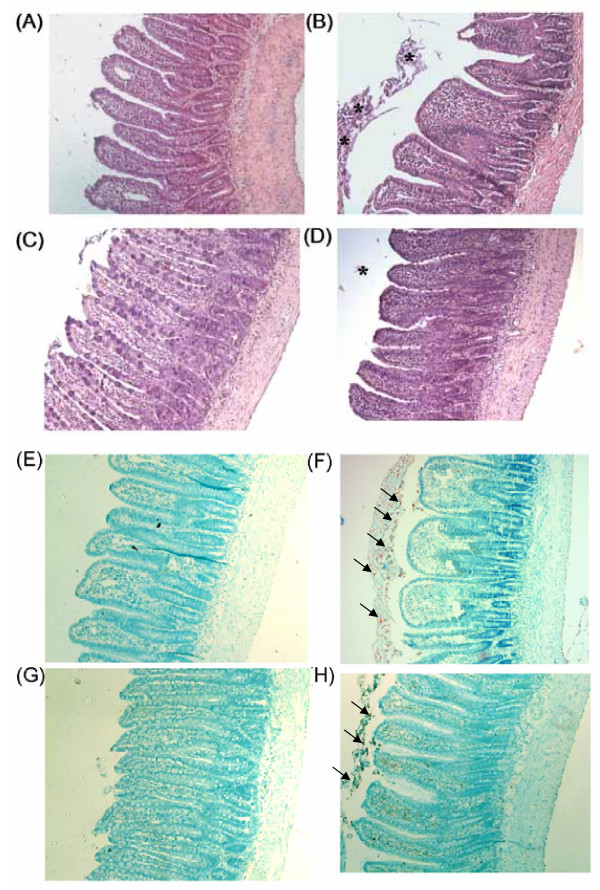
**Histological images of the distal small intestine of sham and IO rats treated with vehicle or ML-7**. **(Panels A to D) **Sections of intestinal tissues were stained with H & E. **(Panels E to H) **The apoptotic level of intestinal tissues was determined by TUNEL assay. All photomicrographs were taken at 100× magnification. Representative images shown were intestinal tissues of four groups of rats: **(A, E) **sham+v, **(B, F) **IO+v, **(C, G) **sham+ML-7, and **(D, H) **IO+ML-7. The IO-induced villous blunting, epithelial shedding (asterisk) and cell apoptosis (arrows) were partially prevented by ML-7, but not by vehicle.

**Figure 7:**
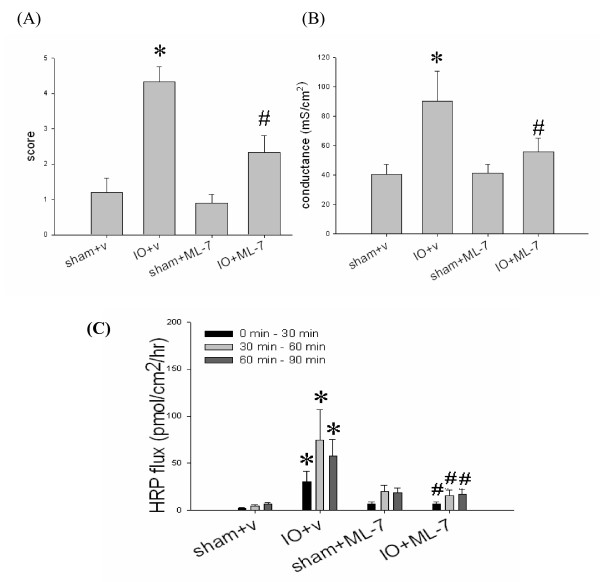
**Administration of ML-7 reduced the histopathological score, and inhibited the epithelial permeability rise in IO rats**. Intestinal tissues of sham and IO rats treated with vehicle (v) or ML-7 were graded for histological damage **(A)**, or mounted on Ussing chambers for the measurement of electrical conductance **(B) **and mucosal-to-serosal HRP fluxes **(C)**. n = 5-7/group. *p < 0.05 vs. sham rats. ^#^p < 0.05 vs. IO+v rats.

The IO-induced mucosal injury, including villous blunting and epithelial sloughing, was reduced by the administration of ML-7 (Figure 6A-D). Increased crypt/villi ratio was seen in IO+v rats compared to sham+v rats (0.970 ± 0.061 vs. 0.667 ± 0.036, p < 0.05), whereas those of IO+ML-7 and sham+ML-7 rats were comparable (0.630 ± 0.025 vs. 0.637 ± 0.059, p > 0.05) (Table 1). The number of apoptotic cells in IO+ML-7 rats was significantly lower than that of IO+v rats (Figure 1G and Figure 6E-H). Treatment of ML-7 decreased the overall histopathological score, and prevented the abnormal increase of conductance and HRP flux caused by IO (Figure 7). Vehicle injection had no effect on these parameters.

**Table 1:** The crypt/villi ratio in the rat distal small intestine.

Group Name	villous height (μm)	crypt depth (μm)	crypt/villi ratio
sham+v	252 ± 11	166 ± 11	0.667 ± 0.036
IO+v	237 ± 13	224 ± 15*	0.970 ± 0.061*
sham+ML-7	251 ± 23	154 ± 8	0.637 ± 0.059
IO+ML-7	283 ± 14	175 ± 13^#^	0.630 ± 0.025^#^

Note. Distal small intestinal tissues from sham and IO rats treated with vehicle (labeled 'sham+v' and 'IO+v') or a MLCK inhibitor, ML-7 (labeled 'sham+ML-7' and 'IO+ML-7') were processed for histological examination. n = 6-7 rats per group. Values were expressed as mean ± SEM. *p < 0.05 vs. respective sham groups. ^#^p < 0.05 vs. IO+v rats.

**Figure 8:**
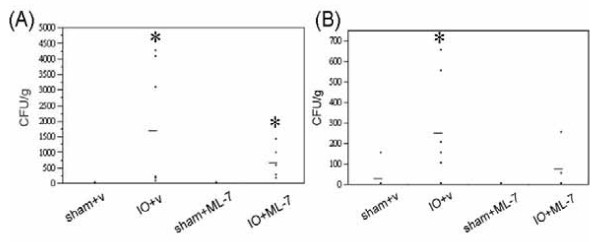
**IO-induced augmentation of BT was not prevented by ML-7**. **(A) **Spleen and **(B) **liver tissues of sham and IO rats treated with vehicle (v) or ML-7 were examined for bacterial growth. Each dot represents the bacterial CFU in the visceral organ of an individual rat. The line indicates the mean for each group. n = 5-7/group. *p < 0.05 vs. respective sham rats.

The mean bacterial CFU in the spleen and liver of IO+ML-7 rats were slightly lower than that of IO+v rats, however, the results did not reach statistical significance (Figure 8). The median scores were also used for nonparametric statistical analysis and showed similar results.

## Discussion and conclusions

Results of the current study demonstrate that bowel obstruction by mechanical ligation evoked mucosal injury and gut barrier damage. The histological damage in our IO rats was similar to other models of mechanical obstruction showing swollen and edematous villous structure [8,24]. The continuity of epithelial layer on the villi was not disrupted in IO rats, in comparison to models with mesenteric ischemia/reperfusion injury that display severe villous denudation and tissue necrosis [9,23]. In consistence with a previous study [8], mucosal damages were only present in close vicinity to the obstructed site, whereas no change was discernible in other parts of the intestine. Moreover, elevated ionic and macromolecular permeability were observed in the distal small intestine and proximal colon, but not in other gut segments, of IO rats.

Enteric bacterial overgrowth in the distal small intestine was associated with an increase in viable bacterial count in the spleen and liver in IO rats. The phenomenon of enhanced BT to extraintestinal organs is consistent with previous reports [8-10]. The most common bacterial species identified in the mesenteric lymph nodes, liver, spleen, and blood samples in bowel obstruction were *Escherichia coli, Klebsiella sp., Bacteroids, Proteus mirabilis, Enterococcus sp*. [4,8]. Results from animal models and patient studies indicated that enteric bacterial overgrowth and microfloral ecological changes may contribute to the influx of enteric bacteria [4,8,25]. Abnormal epithelial permeability and downregulated immunity also played crucial roles in the mechanism of enhanced BT [5,16,26]. We and others showed that IO induced compartmentalized changes in mucosal histology, epithelial permeability and bacterial overgrowth, confined to the distal small intestine. These findings suggested that the distal small intestine could be the major site of bacterial and antigenic influx. Hence we further explored the molecular mechanism responsible for these pathological phenomena.

Excessive epithelial cell apoptosis *in situ *has been suggested to be a causative factor for cell detachment and barrier dysfunction [27-29]. Previous studies demonstrated that inhibition of caspase activation attenuated the increase of epithelial permeability in rodent intestinal transplantation models and in human intestinal cell cultures [29-32]. Our study showed low numbers of apoptotic cells on the villous surface of IO rats and thus, ruled out the possibility that excessive villous apoptotic cell death triggered the enhanced epithelial permeability. The apoptotic cells were found in the intestinal lumen, indicating that IO elicits severe villous epithelial sloughing in which the enterocytes undergo cell death after detachment.

Previous cell culture studies demonstrated that proinflammatory cytokines, e.g. TNFα and IFNγ, disrupted tight junctional structures and upregulated MLCK expression, leading to increase of epithelial permeability [33,34]. We showed that the level of intestinal mucosal TNFα was elevated following IO, which may partly contribute to the induction of intestinal barrier damage. The pharmacological agent (ML-7) was used to decipher the role of MLCK, since ML-7 exerts its inhibitory effect on both isoforms of MLCK by blocking the ATP binding sites on the kinase. MLCK consists of short (130 kD) and long (220 kD) forms. The long form accounts for ~97% of MLCK activity in the intestinal epithelial cells, whereas short MLCK is mainly involved in smooth muscle contraction [35,36]. We confirmed that the phosphorylation level of epithelial MLC was elevated after IO. Addition of ML-7 significantly reduced the epithelial pMLC level in IO rats. The IO-induced rise of tissue conductance and HRP flux were abolished by treatment with ML-7, suggesting an MLCK-dependent increase of paracellular permeability. Recent findings indicated that epithelial MLC phosphorylation not only dilates cell-cell contacts, but also triggers the endocytosis of tight junctional proteins, such as occludin and ZO-1, into specialized apical vacuolar compartments [37,38]. Whether remodeling of tight junctional proteins occurs following bowel obstruction warrants further investigation.

The putative schema for crypt-villus axis involves newly proliferated enterocytes in the crypt base moving toward the villous apex, in which enterocytes mature and differentiate into tight monolayers during the migration process, and ultimately slough off at the 'extrusion zone' [14,39,40]. Accumulating evidence indicated that reorganization of actin filaments and phosphorylation of myosin are required for cell extrusion, and maintenance of epithelial barrier function during physiological cell turnover [14,39-43]. We noticed that the mucosal injury including epithelial shedding was ameliorated in IO+ML-7 rats, indicating an increase in MLCK activation was responsible for premature enterocytic sloughing after bowel obstruction. This forms the first report using an animal model that an MLCK-dependent mechanism is responsible for excessive villous epithelial shedding contributing to imbalance of the intestinal crypt-villus axis.

Lastly, treatment with ML-7 did not significantly decrease the bacterial CFU numbers in the spleen and liver, suggesting that bacterial passage through the obstructed gut may not involve MLCK-dependent paracellular pathways. Administration of ML-7 abolished IO-induced increase of tissue conductance and HRP flux, suggesting that the flux of ions and HRP was mainly through paracellular routes in obstructed guts. However, both paracellular and transcytotic routes may be responsible for the translocation of bacteria across epithelial monolayer. Previous studies demonstrated that under proinflammatory stress and metabolic energy depletion, poorly invasive bacteria were internalized into gut epithelial cells via lipid raft-mediated process and were readily transcytosed [44,45]. Other reports indicated that dilatation of tight junctions and activation of MLCK were associated with BT in cell culture studies and animal models of endotoxemia and stress [15,16,46]. It is speculated that bacterial translocation in IO models may opt for transcellular pathways, and therefore, inhibition by ML-7 did not significantly decrease bacterial influx in obstructed guts. Whether IO triggers transcellular passages of luminal bacteria is currently being studied.

In conclusion, bowel obstruction evoked morphological and functional abnormalities of the gut mucosa, and triggered BT to extraintestinal organs. Increase of the MLCK-dependent epithelial shedding and paracellular permeability were responsible for the elevated flux of macromolecular antigens in the distal small intestine. Moreover, BT may undergo alternative routes other than paracellular passage across the epithelium.

## Abbreviations

IO: intestinal obstruction; MLC: myosin light chain; MLCK: myosin light chain kinase; BT: bacterial translocation; HRP: horseradish peroxidase; CFU: colony forming unit.

## Competing interests

The authors declare that they have no competing interests.

## Authors' contributions

Guarantor of integrity of entire study: LCY; study concepts and design: LCY; data acquisition: CCW, YZL, and LLW; data analysis/interpretation: CCW, and YZL; statistical analysis: CYH; obtained funding: CCW; manuscript drafting or revision for important intellectual content, literature research, manuscript editing, and manuscript final version approval: all authors.

## Pre-publication history

The pre-publication history for this paper can be accessed here:

http://www.biomedcentral.com/1471-230X/10/39/prepub
